# A retrospective study of differences in patients’ anxiety and satisfaction between paper-based and computer-based tools for “Shared Decision-Making”

**DOI:** 10.1038/s41598-023-32448-0

**Published:** 2023-03-30

**Authors:** Jung-Chen Chen, Shang-Feng Tsai, Shih-An Liu

**Affiliations:** 1 Center for Quality Management, Taichung Veterans General Hospital, 1650 Taiwan Boulevard Sect. 4, Taichung, 40705 Taiwan; 2 Division of Nephrology, Taichung Veterans General Hospital, Taichung, 40705 Taiwan; 3 Department of Otolaryngology Head and Neck Surgery, Taichung Veterans General Hospital, Taichung, 40705 Taiwan; 4 Faculty of Medicine, School of Medicine, National Yang Ming Chiao Tung University, Taipei, 11221 Taiwan; 5 Department of Medical Research, China Medical University Hospital, China Medical University, Taichung, 40604 Taiwan

**Keywords:** Patient education, Health policy

## Abstract

We aimed to investigate differences in patients’ anxiety and satisfaction between patients undergoing paper-based patient decision aid (PDA) for shared decision-making (SDM) and those receiving computer-based PDA. We retrospectively collected questionnaires before and after SDM. Basic demographic data as well as anxiety, satisfaction, knowledge acquisition, and participation in SDM were recorded. We divided our population into subgroups according to use of paper-based or computer-based PDA. In addition, Pearson correlation analysis was applied to assess the relationships among variables. In total, 304 patients who visited our Division of Nephrology were included in the final analysis. Overall, over half of the patients felt anxiety (n = 217, 71.4%). Near half of the patients felt a reduction in anxiety after SDM (n = 143, 47.0%) and 281 patients (92.4%) were satisfied with the whole process of SDM. When we divided all the patients based on use of paper-based or computer-based PDA, the reduction of anxiety level was greater in the patients who underwent paper-based PDA when compared with that of those who underwent computer-based PDA. However, there was no significant difference in satisfaction between the two groups. Paper-based PDA was as effective as computer-based PDA. Further studies comparing different types of PDA are warranted to fill the knowledge gaps in the literature.

## Introduction

The World Health Organization Astana Declaration on Primary Healthcare highlights the importance of individual participation in decision-making related to a patient’s healthcare^1^. The patient’s role has altered remarkably during the past few decades and patients are now supposed to be an active partner rather than a passive recipient of healthcare^2^. The National Academy of Medicine (formerly known as the Institute of Medicine) has also stated that patient-centered care plays a crucial role in the delivery of high-quality healthcare. The Committee on Quality of Health Care in America of the National Academy of Medicine describes patient-centered care as a process that comprises “providing care that is respectful of and responsive to individual patient preferences, needs, and values and ensuring that patient values guide all clinical decisions”^3^.

Today, shared decision-making (SDM) is regarded as the supreme model for decision-making by patient representatives, policy-makers, hospitals, and health insurers^4,5^. In addition, SDM is a way in which physicians and their patients join forces to select proper healthcare services based on evidence as well as the patients’ values and preferences^6^. SDM has not only been proposed to be an approach involving mutual respect and participation between doctors and their patients^7^, but has also been reported to improve adherence with medication usage, to enhance the perception of patients’ healthcare-related quality of life, and to reduce the possibility of visiting the emergency room in patients with atherosclerotic cardiovascular disease^8^. Another systematic review and meta-analysis focusing on type 2 diabetes patients found a positive correlation between SDM and better decision quality, patient knowledge, and patient risk awareness^9^.

Patient decision aid (PDA) is designed to facilitate making choices among healthcare decisions and should be evidence-based. It can promote patients’ participation, improve knowledge, and manage expectations about healthcare outcomes^10^. There are two basic types of PDA: paper-based and computer-based. Paper-based PDA is easy to go through quickly and is more feasible to implement in routine clinical practice, whereas computer-based PDA can be presented in different ways and is more flexible as well as providing more information^11^. Patients’ satisfaction with decision-making was improved after SDM^9,12,13^. A previous study comparing the process of SDM between the paper-based and computer-based approaches found that computer-based decision-making aids significantly prolonged the consultation process^14^. However, another study comparing paper and computer-based questionnaires for measuring health outcomes in patients undergoing total hip arthroplasty indicated that use of an electronic form of a questionnaire enabled more efficient and reliable collection of data^15^. Jawaid et al., found that residents’ perception of computer-based assessments was good^16^. Nevertheless, the majority of studies on SDM have been conducted in Western countries^17^ and the preferred model for SDM differs between Chinese and European Americans. Chinese patients preferred family centered decision-making, while European American patients tended to make choices by themselves^18^. In addition, few studies have specifically compared patients’ satisfaction/anxiety level between paper-based and computer-based PDA for SDM. We aimed to investigate differences in patients’ anxiety as well as satisfaction between patients undergoing paper-based PDA and those receiving computer-based PDA in a medical center from a Southeastern Asian country.

## Methods

### Study design

This was a single institute study and the study design was a retrospective chart review. The current study was conducted in accordance with the Declaration of Helsinki and was approved by the Institutional Review Board (IRB) of Taichung Veterans General Hospital (TCVGH) (date: July 27, 2021, approval number: CE21168A). Informed consent was exempted by the IRB of TCVGH as the process had been completed and no interventional procedure was given.

### Material and data source

**Figure 1:**
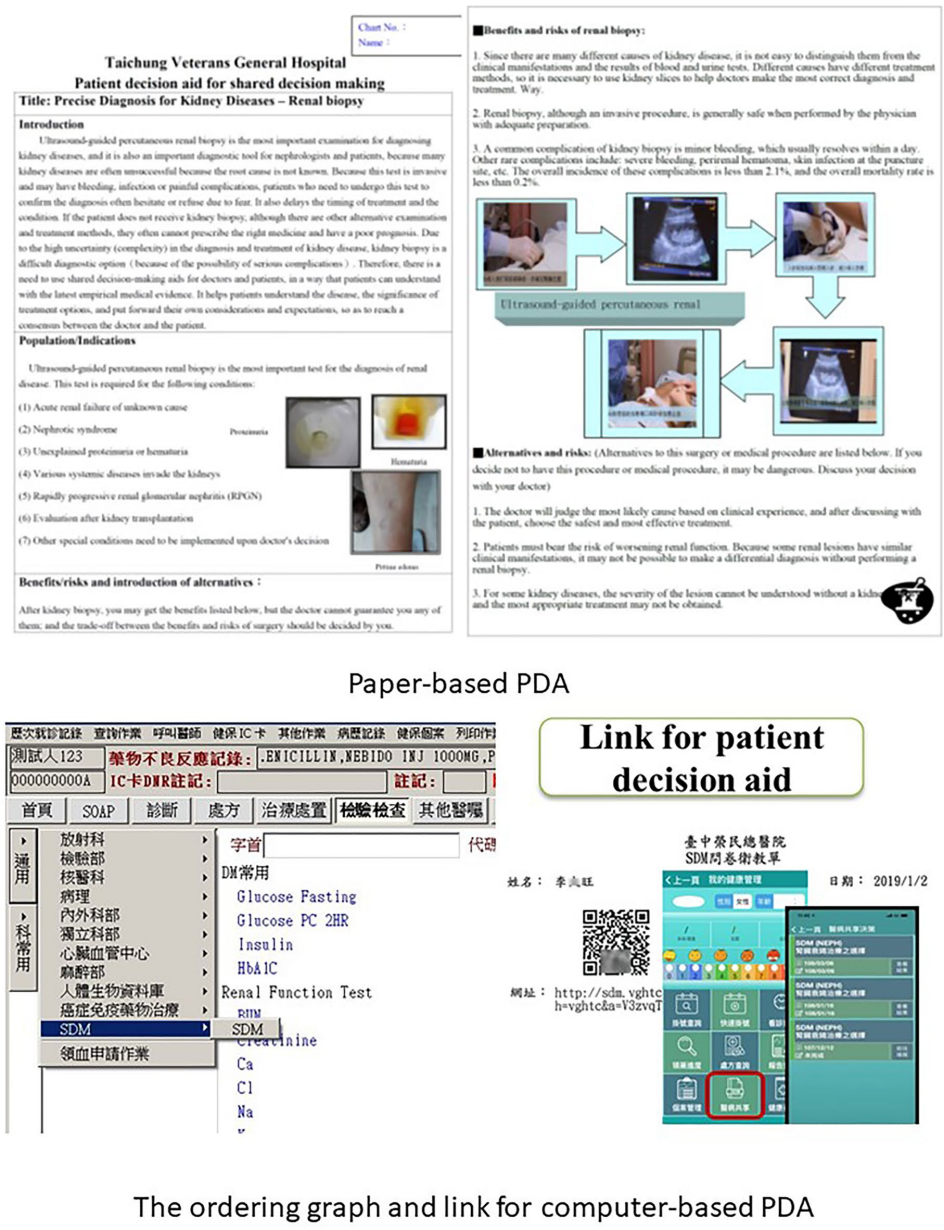
The representative graph for paper-based PDA and the link for computer-based PDA (Should I receive renal biopsy in order to obtain precise diagnosis? Yes, or no?).

The Joint Commission of Taiwan (JCT) has implemented a nationwide SDM program under the support of the Ministry of Health and Welfare since 2016^19^. First, JCT held training courses for clinicians about how to establish and implement SDM in their institutes. Secondly, JCT held competition to select the outstanding teams in developing PDA and publicized these PDAs for other institutes to adopt into their daily practice. Lastly, JCT also held competition to choose the institutes that had distinguished results in implementation of SDM. We sent clinicians to join the training courses held by JCT since the beginning of SDM campaign. Then, we started developed our PDAs since 2017 and PDA for renal biopsy wined the JCT competition in 2018. At the beginning of implementation of SDM, we used paper-based PDA for SDM. Then, we turned paper-based PDA into computer-based PDA since December of 2019. When patients with renal diseases requiring renal biopsy or hemodialysis, clinicians will initiate the process of SDM, which is facilitated by PDA. In addition, we also taught clinician how to use paper-based and computer-based PDA since the launch of SDM campaign. We retrospectively collected the results of questionnaires administered before and after SDM conducted at the Division of Nephrology in TCVGH from May 2017 to June 2021. Besides, the process of SDM was initiated in both in-patient and out-patient settings. Decisions are made after discussion between the patient and physician either via paper-based or computer-based PDA. The topics for SDM include “What type of dialysis should I choose when I have renal insufficiency? Hemodialysis or peritoneal dialysis?” and “Should I receive renal biopsy in order to obtain a precise diagnosis? Yes, or no?”. The questionnaire was developed by the Joint Commission of Taiwan and was widely used by all hospitals in Taiwan implementing SDM. The validity of the questionnaire has been reviewed by experts. In addition, short term test–retest reliability was investigated in the first 30 patients receiving SDM and was considered acceptable with an intraclass correlation coefficient of 0.75. The variables included in the analysis were as follows: age, gender, education, anxiety, satisfaction, knowledge acquisition, whether SDM helped patients know the pros and cons of each decision, whether SDM helped patients know what they care about, whether SDM helped patients make a choice, and participation in SDM. At the beginning of the implementation, paper-based material was used as a decision aid. Paper-based PDA was prepared after critical appraisal of evidence-based medicine and a five-page document was provided to help explain the contents during a routine visit to the clinic. For ease of utilization, we designed an SDM platform that connected patient decision aids with our electronic hospital information system and started using computer-based PDA in December 2019. The information and format presented in the paper and computer-based PDA were similar. The paper-based PDA material and ordering graph as well as the link for the computer-based PDA are presented in Fig. 1. We collected patients’ demographic data as well as anxiety level before receiving both paper-based PDA and computer-based PDA. After SDM, we assessed their anxiety level again and inquired about the abovementioned variables. For ease of analysis, we defined high anxiety as 5 points, moderate anxiety as 4 points, average anxiety as 3 points, slight anxiety as 2 points, and no anxiety at all as 1 point.

### Statistical analysis

We used descriptive statistics to present the demographic data of our patients. For ease of analysis, we divided our population into subgroups according to use of paper-based or computer-based PDA. In addition, the differences in nominal or ordinal variables were analyzed using the Chi-square test. Comparison of anxiety level before and after SDM was examined by paired sample t-test. Pearson correlation analysis was applied to determine the relationships among variables. All analyses were computed by SPSS for Windows, version 22.0 (SPSS, Chicago, IL). The level of statistical significance was set at *P* < 0.05.

### Ethics approval and consent to participate

All methods used in the current study were performed in accordance with the Declaration of Helsinki and the study was approved by the Institutional Review Board (IRB) of Taichung Veterans General Hospital (TCVGH) (date: July 27, 2021, approval number: CE21168A). Informed consent was exempted by the IRB of TCVGH as the process had been completed and no interventional procedure was given.

## Results

### Descriptive results

**Figure 2:**
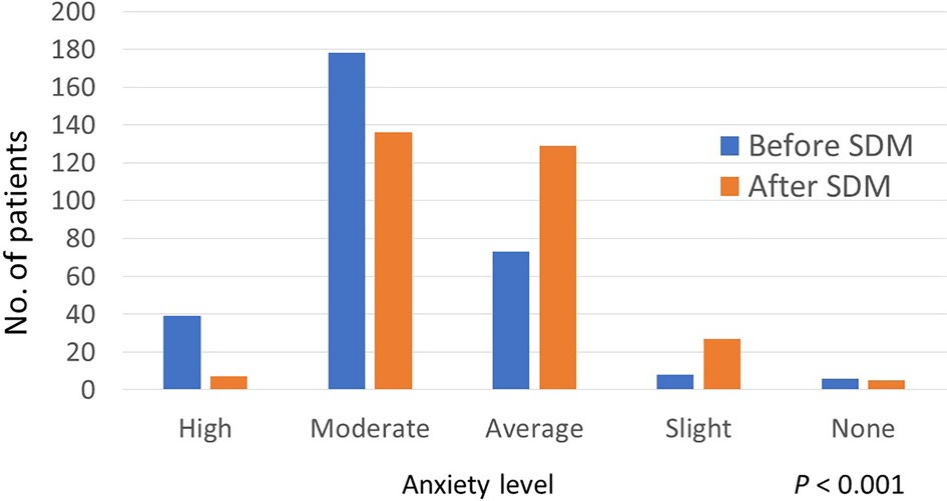
Comparison of anxiety level before and after SDM.

From May 2017 to June 2021, a total of 304 patients who underwent SDM conducted in the Division of Nephrology of TCVGH were enrolled and included in the final analysis. Over half of the participants were female (n = 164, 53.9%), nearly half of the patients were 60 years old or older (n = 145, 47.7%), and over a third of the subjects were 40–59 years old (n = 116, 38.2%). The demographic data are presented in Table 1. In terms of education, high school graduates accounted for the highest proportion of patients (n = 115, 37.8%), followed by patients with elementary school level education (n = 104, 34.2%). Regarding anxiety level before SDM, over half of the patients felt anxiety (n = 217, 71.4%). After implementation of SDM, over half of the patients still could not decide what to choose (n = 159, 52.3%) and 16 patients (5.3%) changed their decision after SDM. Nearly half of the patients recruited their families into the SDM process (n = 142, 46.7%). A similar proportion of patients felt their anxiety was reduced after SDM (n = 143, 47.0%). Two hundred and eighty-one patients (92.4%) were satisfied with the whole process of SDM and 250 patients (82.2%) reported that they had acquired knowledge after SDM. The majority of the patients felt that SDM could improve their knowledge of the pros and cons of each decision (n = 254, 83.6%), as well as their knowledge about what they most care about (n = 248, 81.6%), and making the proper decision (n = 242, 79.6%). Detailed data are presented in Table 2. The patients’ anxiety level reduced markedly after implementation of SDM. When we defined high anxiety as 5 points, moderate anxiety as 4 points, average anxiety as 3 points, slight anxiety as 2 points, no anxiety at all as 1 point, the overall average anxiety scores before and after SDM were 3.78 (± 0.78) and 3.37 (± 0.75), respectively (*P* < 0.001) (Fig. 2).

**Table 1:** Demographic characteristics of patients who underwent shared decision-making (SDM) based on paper-based or computer-based methods.

Variables	Total no. of patients (% in column) (n = 304)	No. of patients (%)		*P* value
		Paper-based (n = 91)	Computer-based (n = 213)	
*Gender*				0.689
Female	164 (53.9%)	47 (28.7%)	117 (71.3%)	
Male	140 (46.1%)	44 (31.4%)	96 (68.6%)	
*Age (years)*				0.001
< 39	43 (14.1%)	16 (62.8%)	27 (37.2%)	
40–59	116 (38.2%)	46 (39.7%)	70 (60.3%)	
≥ 60	145 (47.7%)	29 (20.0%)	116 (80.0%)	
*Education*				0.644
Elementary school	104 (34.2%)	34 (32.7%)	70 (67.3%)	
High school	115 (37.8%)	31 (27.0%)	84 (73.0%)	
University and above	85 (28.0%)	26 (30.6%)	59 (69.4%)	

*SDM* shared decision-making.

**Table 2:** Results of the outcome measures in patients who underwent shared decision-making (SDM) based on paper-based or computer-based methods.

Variables	Total no. of patients (% in column) (n = 304)	No. of patients (%)		*P* value
		Paper-based (n = 91)	Computer-based (n = 213)	
*Anxiety level before SDM*				< 0.001
High	39 (12.8%)	24 (61.5%)	15 (38.5%)	
Moderate	178 (58.6%)	51 (28.7%)	127 (71.3%)	
Average	73 (24.0%)	10 (13.7%)	63 (86.3%)	
Slight	8 (2.6%)	4 (50.0%)	4 (50.0%)	
None	6 (2.0%)	2 (33.3%)	4 (66.7%)	
*Was decision made?*				0.007
Not yet	159 (52.3%)	46 (28.9%)	113 (71.1%)	
Before shared decision-making	38 (12.5%)	4 (10.5%)	34 (89.5%)	
Changes after shared decision-making	16 (5.3%)	4 (25.0%)	12 (75.0%)	
After shared decision-making	91 (29.9%)	37 (40.7%)	54 (59.3%)	
*Anxiety level after SDM*				< 0.001
High	7 (2.3%)	0 (0%)	7 (100%)	
Moderate	136 (44.7%)	7 (5.1%)	129 (94.9%)	
Average	129 (42.4%)	66 (51.2%)	63 (48.8%)	
Slight	27 (8.9%)	16 (59.3%)	11 (40.7%)	
None	5 (1.6%)	2 (40.0%)	3 (60.0%)	
*Satisfaction with SDM*				0.999
Very unsatisfied	0 (0%)	0 (0%)	0 (0%)	
Unsatisfied	3 (1.0%)	1 (33.3%)	2 (66.7%)	
Average	20 (6.6%)	6 (30.0%)	14 (70.0%)	
Satisfied	237 (78.0%)	71 (30.0%)	166 (70.0%)	
Very satisfied	44 (14.5%)	13 (29.5%)	31 (70.5%)	
*Knowledge acquisition after SDM*				< 0.001
Strongly agree	49 (16.1%)	22 (44.9%)	27 (55.1%)	
Agree	201 (66.1%)	40 (19.9%)	161 (80.1%)	
Average	48 (15.8%)	23 (47.9%)	25 (52.1%)	
Disagree	6 (2.0%)	6 (100%)	0 (0%)	
Strongly disagree	0 (0%)	0 (0%)	0 (0%)	
*Difference of anxiety before and after SDM*				< 0.001
Improved	88 (28.9%)	68 (77.3%)	20 (22.7%)	
No change	212 (69.7%)	23 (10.8%)	189 (89.2%)	
Worsened	4 (1.3%)	0 (0%)	4 (100%)	
*Participation in SDM*				0.452
Patient only	162 (53.3%)	45 (27.8%)	117 (72.2%)	
Patient with families	142 (46.7%)	46 (32.4%)	96 (67.6%)	
*SDM helped me know the pros and cons*				< 0.001
Strongly agree	51 (16.8%)	23 (45.1%)	28 (54.9%)	
Agree	203 (66.8%)	39 (19.2%)	164 (80.8%)	
Average	40 (13.2%)	21 (52.5%)	19 (47.5%)	
Disagree	10 (3.3%)	8 (80.0%)	2 (20.0%)	
Strongly disagree	0 (0%)	0 (0%)	0 (0%)	
*SDM helped me know what I care about*				< 0.001
Strongly agree	48 (15.8%)	21 (43.8%)	27 (56.3%)	
Agree	200 (65.8%)	30 (15.0%)	170 (85.0%)	
Average	48 (15.8%)	32 (66.7%)	16 (33.3%)	
Disagree	8 (2.6%)	8 (100%)	0 (0%)	
Strongly disagree	0 (0%)	0 (0%)	0 (0%)	
*SDM helped me make a choice*				< 0.001
Strongly agree	45 (14.8%)	23 (51.1%)	22 (48.9%)	
Agree	196 (64.5%)	27 (13.8%)	169 (86.2%)	
Average	60 (19.7%)	38 (63.3%)	22 (36.7%)	
Disagree	3 (1.0%)	3 (100%)	0 (0%)	
Strongly disagree	0 (0%)	0 (0%)	0 (0%)	

*SDM* shared decision-making.

### Subgroups analysis

When we stratified our patients based on paper-based and computer-based PDA, there were no significant differences between these two groups in gender, education, satisfaction with SDM, and participation in SDM. However, patients receiving computer-based PDA were older, had a lower anxiety level before SDM, made more decisions before SDM, had more knowledge acquisition after SDM, had limited difference in anxiety before and after SDM, and had a more positive feeling about the help they received in terms of knowing the pros and cons/knowing what they care about/helping them make a choice. Detailed data are shown in Table 2.

**Figure 3:**
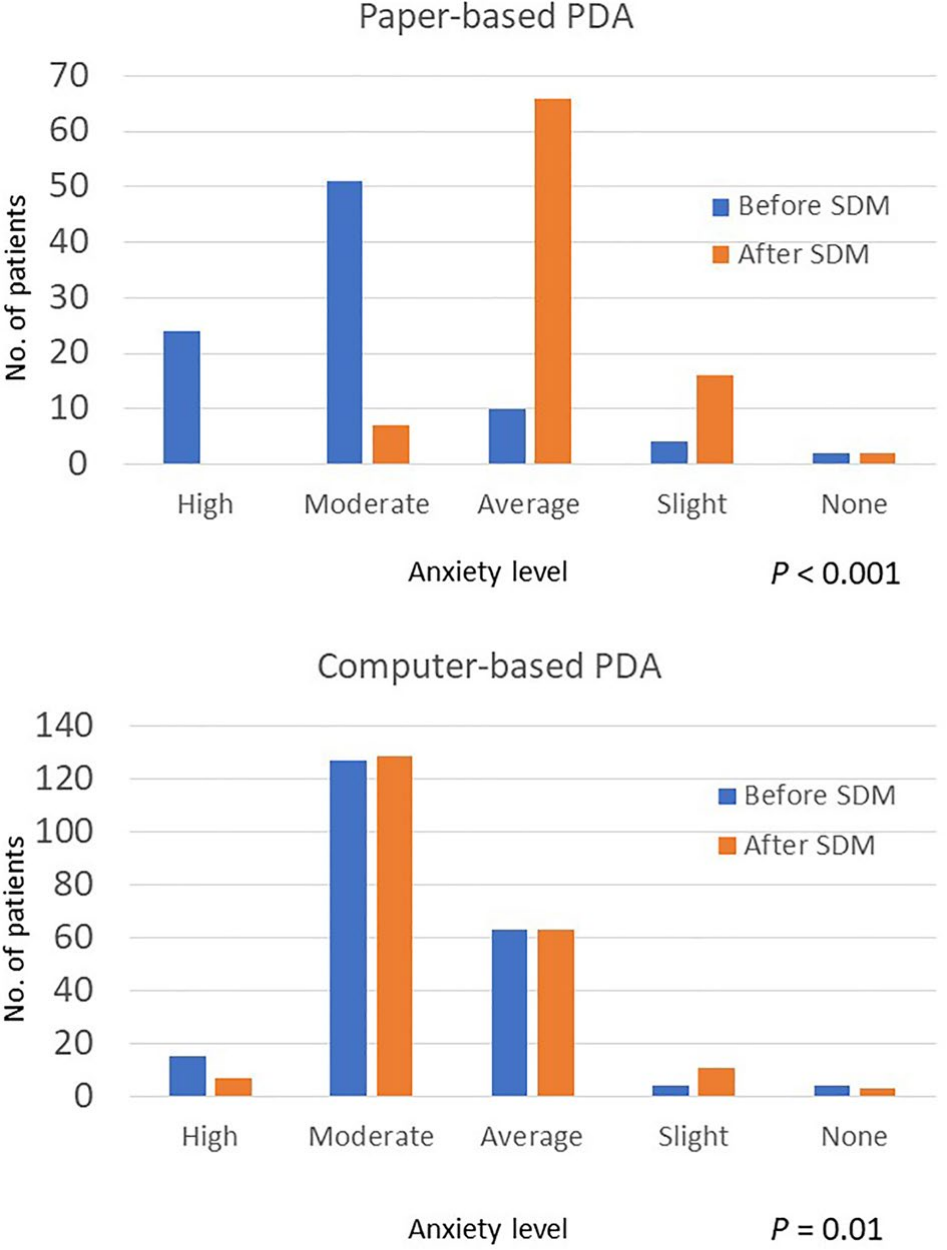
Comparison of anxiety level before and after SDM based on paper-based or computer-based.

When we divided all patients according to paper-based or computer-based PDA, the reduction of anxiety level was greater in patients who underwent paper-based PDA (before SDM: 4.00 ± 0.87; after SDM: 2.86 ± 0.57, respectively, *P* < 0.001) when compared with those who underwent computer-based PDA (before SDM: 3.68 ± 0.72; after SDM: 3.59 ± 0.71, respectively, *P* = 0.01) (Fig. 3).

### Pearson correlation analysis

The correlation coefficients among the variables are presented in Table 3. A higher anxiety level before SDM was positively associated with “decision already made before SDM” and more knowledge acquisition. In addition, female gender was associated with a higher level of anxiety before SDM, more knowledge acquisition, and “decision already-made before SDM”. A higher education level was related to “decision already-made before SDM” and “decision was made without family”. Furthermore, family-involved SDM seemed to be related to less knowledge acquisition, and more knowledge acquisition was strongly associated with satisfaction with SDM. In contrast, “decision not yet made before SDM” was associated with greater satisfaction with SDM.

**Table 3:** Pearson correlation coefficients of the variables involved in the shared decision-making process.

	Gender	Age	Education	Anxiety before SDM	Was decision made	Knowledge acquisition	Participation in SDM
Age	0.121 **0.035**						
Education	0.048 0.402	− 0.604 ** < 0.001**					
Anxiety before SDM	− 0.82 0.152	− 0.007 0.902	0.026 0.656				
Was decision made?	− 0.136 **0.018**	− 0.086 0.134	0.146 **0.011**	0.232 ** < 0.001**			
Knowledge acquisition	− 0.146 **0.011**	0.061 0.289	− 0.055 0.337	0.165 **0.004**	0.081 0.157		
Participation in SDM	− 0.85 0.141	0.272 ** < 0.001**	− 0.186 **0.001**	0.006 0.910	0.004 0.947	− 0.179 **0.002**	
Satisfaction with SDM	− 0.30 0.597	0.028 0.632	− 0.007 0.898	0.103 0.074	− 0.187 **0.001**	0.470 ** < 0.001**	0.101 0.079

Gender: 1: male; 0: female.

Age: 1: < 39 years; 2: 40–59 years; 3: ≥ 60 years.

Education: 1: Elementary school; 2: high school; 3: College/university and above.

Anxiety level before SDM: 1: None; 2: Slight; 3: Average; 4: Moderate; 5: High.

Was decision made: 1: Not yet; 2: Yes.

Knowledge acquisition: 1: Strongly disagree; 2: Disagree; 3: Average; 4: Agree; 5: Strongly agree.

Participation in SDM: 1: Patient only; 2: With family.

Satisfaction with SDM: 1: Very unsatisfied; 2: Unsatisfied; 3: Average; 4: Satisfied; 5: Very satisfied.

Significant *P* values are in bold.

## Discussion

This is the first study comparing the effect of paper-based and computer-based PDA on anxiety level before and after implementation of SDM from a Southeast Asian country. We found anxiety level reduced significantly after SDM, especially in those who used paper-based PDA. A previous review article indicated that physician empathy was strongly linked to the reduction of patients’ anxiety^20^. In addition, Waldron et al., in their research also indicated a power imbalance inevitably existed between healthcare professionals and patients^21^. Furthermore, patients who acquired emotional reassurance from their doctors were less anxious and more satisfied^22^. This could explain why the anxiety level of our patients reduced significantly after SDM. However, we found that computer-based PDA was not as effective as paper-based PDA at reducing anxiety level. A previous study found that computer-based decision aids significantly lengthened consultations and doctors acted largely as information providers for the patients^13^. As time is one of the proposed barriers in the practice of SDM, doctors might have less time to have a discussion with patients when using a computer-based decision aid. In addition, Werner et al., found that younger people had less anxiety using a computer^22^. As mentioned in the results section, the patients receiving computer-based PDA in our study were older than those who used paper-based PDA. The abovementioned reasons could explain why computer-based PDA was not as effective as paper-based PDA for reducing anxiety level.

It is interesting to note that there was no significant difference in patients’ satisfaction between patients undergoing paper-based PDA and those receiving computer-based PDA, even though there was a greater reduction in anxiety level after SDM in patients receiving paper-based PDA. Many previous studies revealed that SDM was related to better patient satisfaction^3,7,12,23^. A methodological review found that there were no significant differences in data integrity, time to complete survey, or data consistency among diverse survey questionnaires methods including paper-based, laptop computer-based, tablet computer-based, tablet app, and short message service^24^. In another study that compared paper- and computer-based questionnaire modes of measuring health outcomes in patients undergoing total hip arthroplasty, no significant differences were detected between the two methods in all of the questionnaire items^15^. Again, physician empathy was strongly linked to the reduction of patients’ anxiety, and patients who acquired emotional reassurance from their doctors were less anxious and more satisfied^20,23^. Moreover, the satisfaction rates were relatively high in both groups (paper-based vs. computer-based: 92.3% vs. 92.5%). These reasons probably explain why anxiety level improved and yet satisfaction remained the same in the current study. Nevertheless, Tsai et al.^25^ investigated implementation of a patient-centered mobile SDM platform and healthcare workers’ evaluation and found that mobile SDM offers patients and their families an easy way to address their concern to healthcare professionals and improves their relationship with each other meaningfully. Use of survey questionnaires with an electronic format facilitates more efficient and reliable data collection^15^. Therefore, we still recommend using electronic forms during the SDM process.

Gender was reported to affect patients’ perceptions of the importance of participation^2^. However, few studies have addressed the impact of gender on the process of SDM. The current study found females tended to make a decision before SDM and had more knowledge acquisition after SDM. The reasons for this phenomenon warrant further studies and the design of SDM should address this issue. In addition, those with less education were less informed and the clinician was more likely to make a decision rather than the patients themselves^26^. This could explain why patients with a higher education level tended to make a decision by themselves before SDM in the current study.

Nearly half of our participants made their decision with their families. Previous studies mentioned that families were less involved in SDM^17,27,28^. However, family involvement differs based on the type of illness, the treatment choice and the patients’ culture^28^. A previous study noted that Chinese valued family-centered decision-making more than European Americans. However, ethnic Chinese living in America seemed to prefer traditional SDM to the same extent as European Americans^18^. Nevertheless, as most studies on SDM originated from Western countries, research from non-Western countries is warranted to better understand cultural issues related to SDM^17^. In our study, we included more patients underwent computer-based PDA when compared with those receiving paper-based PDA. There are three reasons to explain such phenomenon. First, in the beginning of SDM campaign, clinicians were not familiar with the processes. Secondly, paper-based PDA might be lost during collection. Lastly, there were missing answers in paper-based PDA and we must discard such data in final analysis (computer-based PDA would check the completeness of the questionnaire automatically).

The limitations of the current study were as follows. First, this was a single institute study and the external validity of our findings was insufficient. Second, we only included patients with nephrotic problem. Moreover, the study design was retrospective, and therefore it was not bias-free. Lastly, this was not a randomized control trial, so selection bias inevitably existed and we did not perform sample size calculation in advance.

## Conclusion

According to the results of the current study, paper-based and computer-based PDA can reduce anxiety levels after implementation of SDM, and paper-based PDA seemed to be better in this regard. In addition, the patients’ levels of satisfaction were both high no matter which format was adopted during SDM. Further studies comparing patients’ perception/satisfaction among different types of PDA are warranted to fill the knowledge gaps in the literature.

## Supplementary Information

Supplementary Information.

## Data Availability

All data generated or analyzed during the current study are available from the corresponding author on reasonable request.
