# Supplementary material for: A retrospective study of differences in patients’ anxiety and satisfaction between paper-based and computer-based tools for “Shared Decision-Making”
Source: Sci Rep. 2023 Mar 30;13:5187. doi: 10.1038/s41598-023-32448-0 (PMC10063635; doi:10.1038/s41598-023-32448-0)
Supplement: Supplementary file 1 — Supplementary Information. [file 41598_2023_32448_MOESM1_ESM.pdf]

Questionnaire for patients receiving share decision-making

Basic Information of the Respondent

A. Respondent is: 1.☐ Patient 2.☐ Patient's families

B. Gender: 1.☐ Male 2.☐ Female

C. Age:

1.☐ ≤19 years 2.☐ 20-29 years 3.☐ 30-39 years 4.☐ 40-49 years
5.☐ 50-59 years 6.☐ 60-69 years 7.☐ > 65years

D. Education:

1.☐ None 2.☐ Elementary school 3.☐ Junior high school 4.☐ Senior high school
5.☐ College/University 6.☐ Master degree 7.☐ PhD

E. The person who looks at the PDA and involves in decision-making is:

1.☐ Myself 2.☐ Parents 3.☐ Spouse 4.☐ Children 5.☐ Others: ______

F. Your level of anxiety in the face of current medical issues before making a shared decision?

1.☐ None 2.☐ Slight 3.☐ Average 4.☐ Moderate 5.☐ High

G. Was decision made before shared decision making? 1. ☐ Not yet 2. ☐ Yes

**Can this decision aid...**		1: Strongly disagree; 2: Disagree; 3: Average; 4: Agree; 5: Strongly agree				
1	Helps you recognize that you have to make a decision?	1	2	3	4	5
2	Helps you to make a choice?	1	2	3	4	5
3	Helps you know the pros and cons of each decision?	1	2	3	4	5
4	Helps you think about which pros and cons are most important?	1	2	3	4	5
5	Help you know what you care about the most?	1	2	3	4	5
6	Help you organize your own thoughts about these decisions?	1	2	3	4	5
7	Help you think about how involved you can be in this decision?	1	2	3	4	5
8	Help you identify questions you want to ask your doctor?	1	2	3	4	5
9	Getting you ready to tell your doctor what's on your mind?	1	2	3	4	5
10	Getting you ready for a follow-up with your doctor?	1	2	3	4	5
11	Are there any descriptions, pictures, presentation methods or questions in this decision aid that make it difficult for you to understand or answer? Please briefly describe:______________					
12	Do you have a decision-related question you want to know, but this decision aid doesn't address? Please briefly describe:____					

H. Your level of anxiety in the face of current medical issues after making a shared decision?

1.☐ None 2.☐ Slight 3.☐ Average 4.☐ Moderate 5.☐ High

I. Your satisfaction about the process of shared decision making.

1.☐ Very unsatisfied 2.☐ Unsatisfied 3.☐ Average 4.☐ Satisfied 5.☐ Very satisfied
